# Serum IGFBP-1 as a promising diagnostic and prognostic biomarker for colorectal cancer

**DOI:** 10.1038/s41598-024-52220-2

**Published:** 2024-01-22

**Authors:** Bin-Liang Huang, Lai-Feng Wei, Yi-Wei Lin, Li-Sheng Huang, Qi-Qi Qu, Xin-Hao Li, Ling-Yu Chu, Yi-Wei Xu, Wei-Dong Wang, Yu-Hui Peng, Fang-Cai Wu

**Affiliations:** 1 Department of Clinical Laboratory Medicine, The Cancer Hospital of Shantou University Medical College, Shantou, China; 2 Precision Medicine Research Center, Shantou University Medical College, Shantou, China; 3 Department of Clinical Laboratory Medicine, Jiangmen Central Hospital, Affiliated Jiangmen Hospital of Sun Yat-Sen University, Jiangmen, China; 4 Department of Radiation Oncology, The Cancer Hospital of Shantou University Medical College, 7 Raoping Road, Shantou, 515041 China; 5 Department of Bone and Soft Tissue Oncology Surgery, The Cancer Hospital of Shantou University Medical College, Shantou, China; 6 Guangdong Esophageal Cancer Institute, Guangzhou, China

**Keywords:** Cancer, Gastrointestinal cancer

## Abstract

Our previous study showed that levels of circulating insulin-like growth factor binding protein-1 (IGFBP-1) has potential diagnostic value for early-stage upper gastrointestinal cancers. This study aimed to assess whether serum IGFBP-1 is a potential diagnostic and prognostic biomarker for CRC patients. IGFBP-1 mRNA expression profile data of peripheral blood in colorectal cancer (CRC) patients were downloaded and analyzed from Gene Expression Omnibus database. We detected serum IGFBP-1 in 138 CRC patients and 190 normal controls using enzyme-linked immunosorbent assay. Blood IGFBP-1 mRNA levels were higher in CRC patients than those in normal controls (*P* = 0.027). In addition, serum IGFBP-1 protein levels in the CRC group were significantly higher than those in normal control group (*P* < 0.0001). Serum IGFBP-1 demonstrated better diagnostic accuracy for all CRC and early-stage CRC, respectively, when compared with carcinoembryonic antigen (CEA), carbohydrate antigen19-9 (CA 19-9) or the combination of CEA and CA19-9. Furthermore, Cox multivariate analysis revealed that serum IGFBP-1 was an independent prognostic factor for OS (HR = 2.043, *P* = 0.045). Our study demonstrated that serum IGFBP-1 might be a potential biomarker for the diagnosis and prognosis of CRC. In addition, the nomogram might be helpful to predict the prognosis of CRC.

## Introduction

CRC is one of the most common gastrointestinal malignancies with high prevalence and mortality. It is the third leading cause of cancer and the second leading cause of cancer-related death in the world^1,2^. It is estimated that 1.93 million new CRC cases and 940,000 deaths occurred in 2020, accounting for about 1 in 10 cancer cases and deaths worldwide^1^. In the past decades, with the changes in people’s lifestyles and dietary habits, the incidence and mortality of CRC in China have risen increasingly^3^, and males in urban areas have a higher incidence^4^. Most patients with CRC are asymptomatic in the early stage, and are often diagnosed in the advanced stage or even accompanied by distant metastasis when clinical symptoms appear, which usually results in a poor prognosis for most patients^5^. It is generally believed that CRC is curable and preventable, especially in the early stage^6^. Using colonoscopy in CRC screening can detect precancerous polyps and small cancerous lesions that can be excised directly during the procedure, thereby decreasing the incidence of CRC to some extent^7–9^. And survival rate could be significantly improved when patients are diagnosed at an early stage^10,11^. Additionally, some other CRC early screening methods including fecal occult blood testing (FOBT), fecal immunochemical tests (FITs) and stool DNA test were carried out to reduce the incidence and mortality of CRC^12^. However, the widespread use of these methods in the populations is restricted due to its invasive nature or low specificity/sensitivity^13–16^.

It is clear that the improvement in the survival of CRC patients is limited owing to the limitation of early detection, prognostic assessment and decision-making capacity for optimal treatment. Reliable biomarkers with high specificity and sensitivity are expected to be helpful for early diagnosis, prognostic evaluation, and the prediction of treatment response and recurrence risk for CRC^17^. Equally important, circulating protein biomarkers have been widely investigated as minimal invasive diagnostic and prognostic strategies for CRC^18–21^, including CEA, CA 19-9, p53, and vascular endothelial growth factor (VEGF)^21,22^. Of these biomarkers, CEA and CA19-9 have been the most studied because of their impact on the diagnosis and prognosis of CRC patients, but their specificity and sensitivity at early diagnosis are limited^23,24^. The best strategy for early diagnosis and prognostic assessment of CRC should be reliable, economically feasible, and to which patients can adhere over the long term. Up to now, there is still insufficient evidence to determine which approach is absolutely superior for the diagnosis and prognosis of CRC. Therefore, seeking more reliable tools to identify patients with early-stage CRC and to assess the prognosis of patients is the key to effective treatment and improvement in the survival of CRC patients.

IGFBP-1 is one of the key members of the insulin-like growth factor (IGF) system that plays a vital role in growth/development, metabolism and the pathophysiology of cancers^25^. IGFBP-1 can function by activating cell-surface receptors directly in an IGF-independent way, which can enhance the migration or adhesion ability for cancer cells^26–28^. In addition, as a secreted protein, aberrant expression in circulating and potential clinical implications of IGFBP-1 in patients with cancer have been reported^29,30^. In our previous studies, we found that serum IGFBP-1 levels were significantly increased in upper gastrointestinal cancers, and could distinguish early-stage upper gastrointestinal tumors with high diagnostic accuracy^31^. However, the diagnostic and prognostic value of serum IGFBP-1 in patients with CRC has not yet been examined. In this study, we aimed to investigate IGFBP-1 levels in CRC and evaluate whether IGFBP-1 could be served as a diagnostic and prognostic biomarker in patients with CRC.

## Materials and methods

### Study participants

In this study, 138 serum samples of patients with CRC and 190 serum samples of normal controls were collected from the Cancer Hospital of Shantou University Medical College between June 2013 and May 2014. Samples from patients with CRC collected in this study met the following eligibility criteria: (1) they were all newly diagnosed CRC patients by histopathology without any anticancer treatment before blood collection; (2) they had complete baseline clinical information and complete follow-up data; (3) they had no history of cancer, type 1 and type 2 diabetes or cardiovascular diseases. The normal controls were from those who were identified by health check-up, and all of them did not suffer from any cancer diseases and diabetes or cardiovascular disease.

This study was performed after approval from the Ethics Committee of the Cancer Hospital of Shantou University Medical College (approval number 201901), and informed consents were obtained from all participants. This work complied with the principles laid down in the Declaration of Helsinki. In this study, the 8th edition of the American Joint Committee on Cancer (AJCC) cancer stage manual was adopted^32^. We classified tumors with AJCC stage 0+I+II as early-stage CRC. The demographic details and clinicopathological data of all patients with CRC were recorded, such as age, gender, smoking behavior, drinking behavior, depth of tumor invasion, lymph node metastasis, distant metastasis, and tumor node metastasis (TNM) stage.

### Collection of blood samples

Peripheral blood samples of CRC patients and normal controls were processed in an identical manner, which were collected into anticoagulant-free tubes and coagulated at room temperature for 30 min, centrifuged at 1250* g* for 10 min, and then stored at − 80 °C until the beginning of the experiment.

### GEO expression profiling data analysis

The expression profiling data from the peripheral blood of CRC patients and the corresponding clinical data were obtained from the GEO database (https://www.ncbi.nlm.nih.gov/geo/). We selected a set of GEO expression spectrum data: GSE164191.

### ELISA for IGFBP-1

The expression levels of serum IGFBP-1 were measured by a commercial ELISA kit (CUSABIO, Wuhan, China), and the procedure was carried out according to the manufacturer's recommendations in the ELISA kit. Briefly, 100 μl of serum sample at 20 fold-dilution and standard were added into antibody-coated 96-microwell plate, which were covered with the adhesive strip and then incubated for 2 h at 37 °C. Then, the liquid of each well was poured out, followed by the addition of 100 μl biotin-antibody (1X) in each well for 1 h at 37 °C. After removing the liquid and washing with wash buffer, 100ul horseradish peroxidase (HRP)-avidin (1X) was added to each well and incubated for 1 h at 37 °C. After washing the plate, TMB substrate was added into the microplates and incubated for 15–30 min in a dark environment for color development, and stop solution was used to stop the reaction. The optical density (OD) values were read at 450 and referenced to 570 nm wavelengths within 5 min after adding stop solution by microplate reader (Thermo Fisher Scientific, Boston, USA).

The concentrations of serum IGFBP-1 were obtained by plotting a standard curve with a four-parameter logistic curve manner, and actual serum IGFBP-1 concentration must be multiplied by the dilution factor. Serum samples of patients and normal controls were measured simultaneously in the same batch. All measurements were done in duplicate.

### Measurement of tumor markers in clinical use

The concentrations of serum CEA and CA19-9 were measured by an automatic electrochemical luminescence analyzer (Cobas e601, Roche, Germany). All tests of the two tumor markers were performed at the Department of Clinical Laboratory Medicine, the Cancer Hospital of Shantou University Medical College, and operated according to the instrument operating manual. In this study, the recommended clinical cutoff values of CEA, CA19-9 were 5.0 ng/mL and 27 U/mL, respectively.

### Statistical analysis

Statistical analysis was performed using SPSS software (version 22.0), GraphPad Prism software (version 8.0), and Microsoft Excel. We used the Mann–Whitney U test or t-test to compare the differences of IGFBP-1 expression between CRC and control groups. All serum IGFBP-1 expression data are exhibited as means ± standard deviations (SDs). The correlation between positive rates of serum IGFBP-1 and clinicopathological features was analyzed by means of Chi-squared test. The receiver operating characteristic (ROC) curves were used to assess the diagnostic value of serum IGFBP-1 in the identification of CRC patients and normal controls and evaluate sensitivity, specificity, and AUCs with 95% confidence interval (CI). As previously described, the optimum cutoff value for IGFBP-1 levels was obtained by achieving the maximum sensitivity when the specificity was > 90%, and by minimizing the distance of the cut-off value to the top-left corner in the ROC curve^31^, which could contribute to produce an economical, feasible tests, and make a benefit to early cancer detection^33^. Subsequently, positive predictive values (PPV), negative predictive values (NPV), positive likelihood ratio (PLR), and negative likelihood ratio (NLR) were calculated by mean of these optimal cutoff values.

We used X-tile to classify CRC patients with higher or lower serum IGFBP-1 levels and then the best cut-off value was obtained. The OS of patients was calculated by the Kaplan–Meier method, and the significant difference was evaluated by the log-rank test. Univariate and multivariate Cox regression analyses were performed to identify independent risk factors for CRC prognosis. A nomogram was established using all variables with a *P*-value < 0.05 in multivariate Cox regression by the package of *rms* in R. In addition, the associated calibration curves were applied to assess the predictive ability of 1-, 3- and 5 years OS of the nomogram. The predictive accuracy and discriminative ability of the nomogram were assessed by C-index and decision curve analysis (DCA). In this study, the terminal point was OS, which was defined as the time interval from the date of receiving surgery to any form of death, and the data were censored for patients who were alive at the date of the last follow-up of June 2022. In all statistical tests, we considered *P*-values (two sided) of less than 0.05 to be statistically significant.

## Results

### Peripheral blood IGFBP-1 mRNA levels in patients with CRC

In order to investigate the expression of IGFBP-1 in patients with CRC. The expression profiling data from the peripheral blood of CRC patients and normal controls were obtained from the GEO database (GSE164191). In this dataset, A total of 120 participants were enrolled, including 59 patients with CRC and 61 healthy patients. After processing data, statistical analysis found that IGFBP-1 mRNA expression level was up-regulated in patients group compared to normal controls group (*P* = 0.027, Supplementary Fig. S1A,B). These results demonstrated that blood levels of IGFBP-1 mRNA increase in patients with CRC.

### The level of serum IGFBP-1 in CRC patients and normal controls

**Figure 1:**
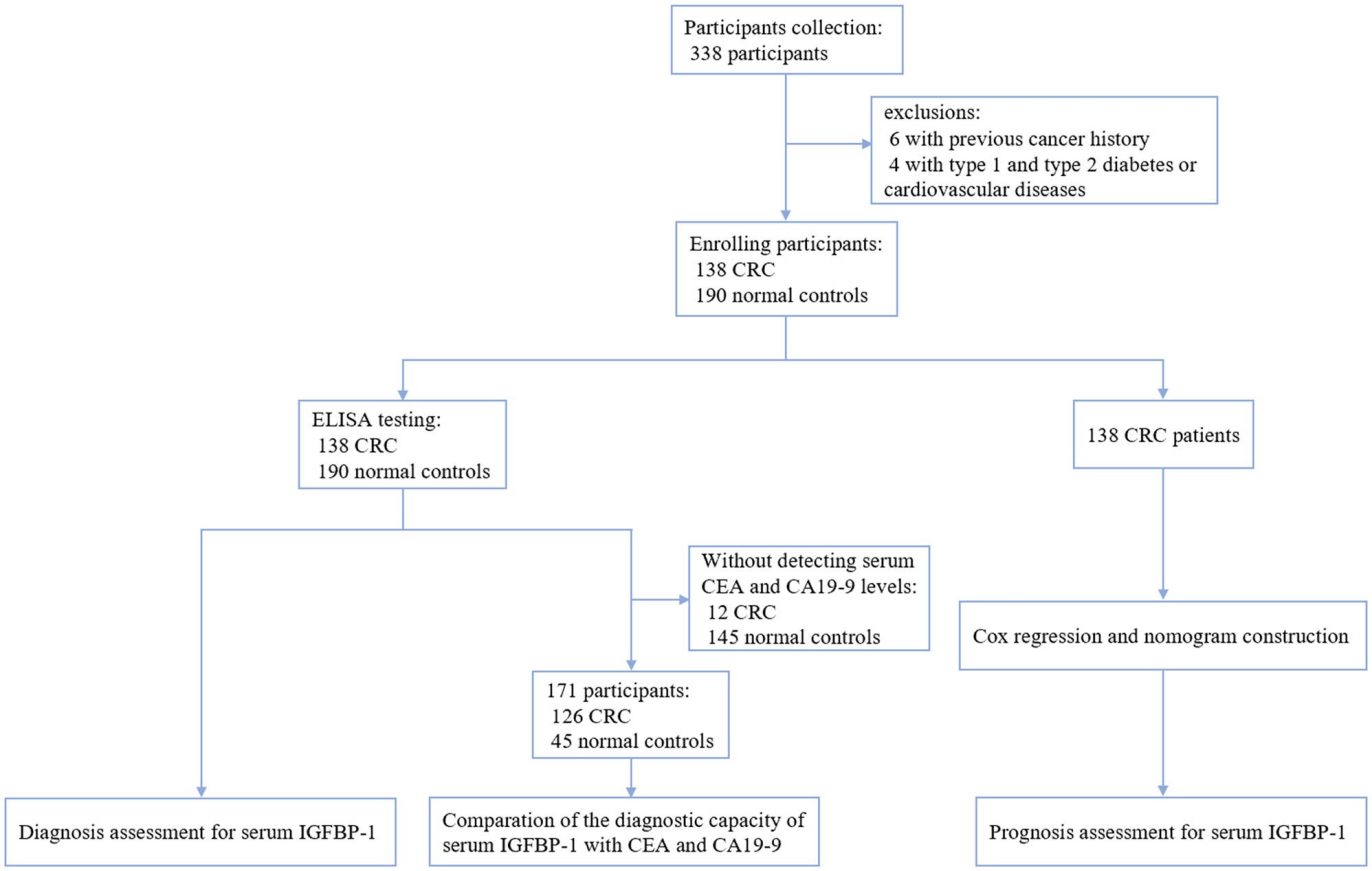
Study overview of serum IGFBP-1 in colorectal cancer.

**Figure 2:**
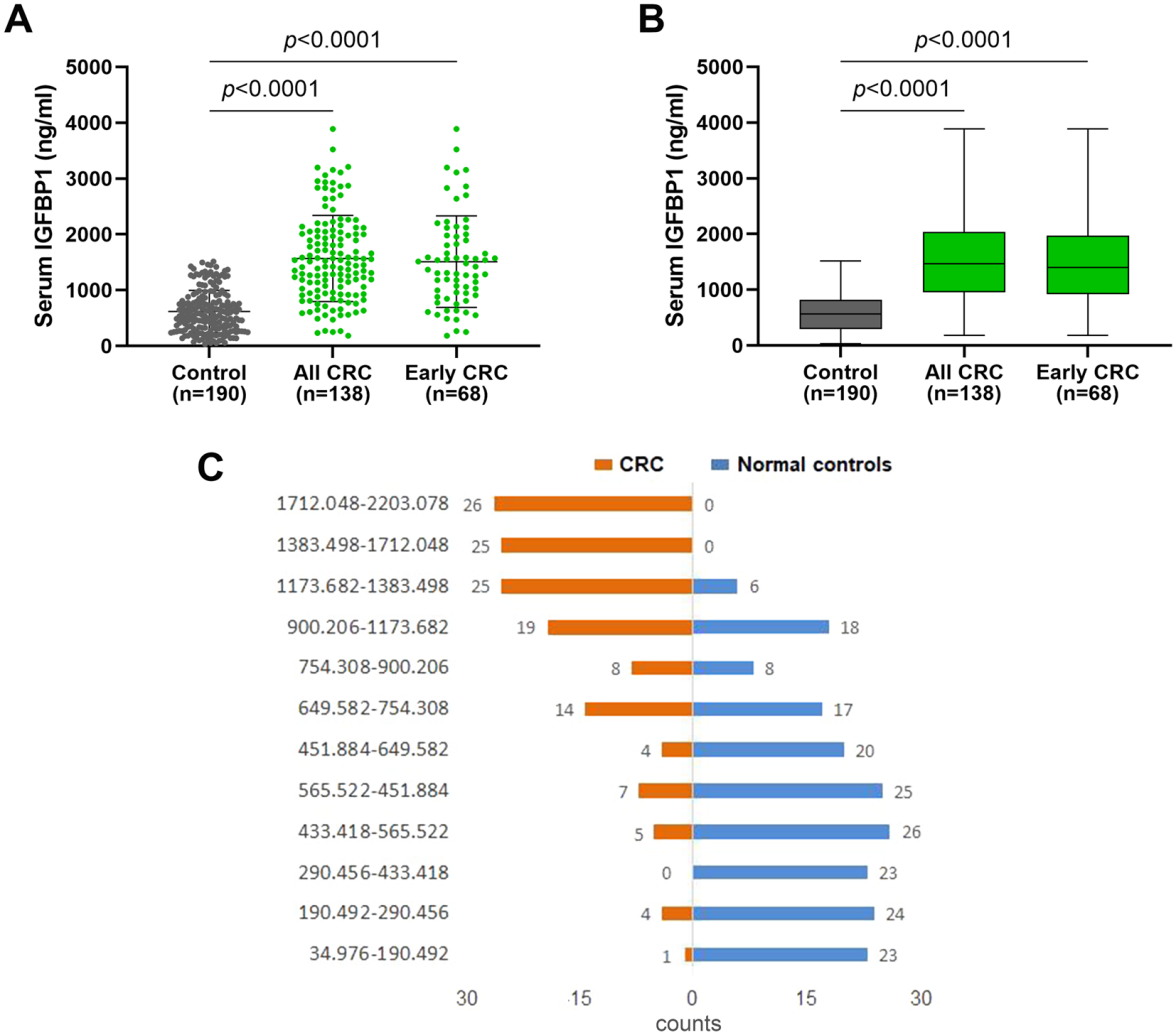
The levels of serum IGFBP-1protein in CRC patients and normal controls. (**A**) The expression of serum IGFBP-1 of every sample in three groups was shown in scatter plot and box plot (*P* < 0.0001). Black horizontal lines are means, and error bars are SEs. (**B**) The box plot showed the degree of dispersion. The line in the box is the median. (**C**) The lowest concentration was 34.976 ng/mL in normal controls and the highest concentration was 2203.078 ng/mL in CRC. The diagram of CRC is in orange and normal controls is in blue. CRC accounts for more of the histogram volume on higher concentration, but normal controls accounts for more lower concentration.

Subsequently, we performed ELISA to further evaluate serum levels of IGFBP-1 in CRC patients and normal controls. In total, 328 participants were recruited, 138 patients with CRC and 190 normal controls (Fig. 1). As shown in Supplementary Table S1, the mean age of 138 eligible patients was 58 years (range 26–82 years), of which 78 (56.5%) were males and 60 (43.5%) were females. The control group consisted of 147 (77.37%) males and 43 (22.63%) females aged between 40 and 80 years (mean, 56 years). The mean concentration of serum IGFBP-1 was 1569.455 ± 770.209 ng/mL, 1512.222 ± 818.971 ng/mL and 719.991 ± 379.340 ng/mL in CRC group (n = 138), early-stage CRC group (n = 68) and normal group (n = 190), respectively (Supplementary Table S2). To get a better view of its distribution and degree of dispersion, the levels of serum IGFBP-1 in three groups were shown in scatter plot (Fig. 2A) and box plot (Fig. 2B). The levels of serum IGFBP-1 in CRC group were higher when compared with normal group, which was confirmed statistically (*P* < 0.0001). In addition, the difference between early-stage CRC and normal controls is also significant (*P* < 0.0001). It can be observed that the distribution of CRC and normal controls is different in Fig. 2C. CRC group accounts for more histogram volume on higher concentration while normal group for more lower concentration. In a word, our data demonstrated that serum levels of IGFBP-1 significantly increase in CRC patients.

### The diagnostic value of serum IGFBP-1 in CRC and early-stage CRC

**Figure 3:**
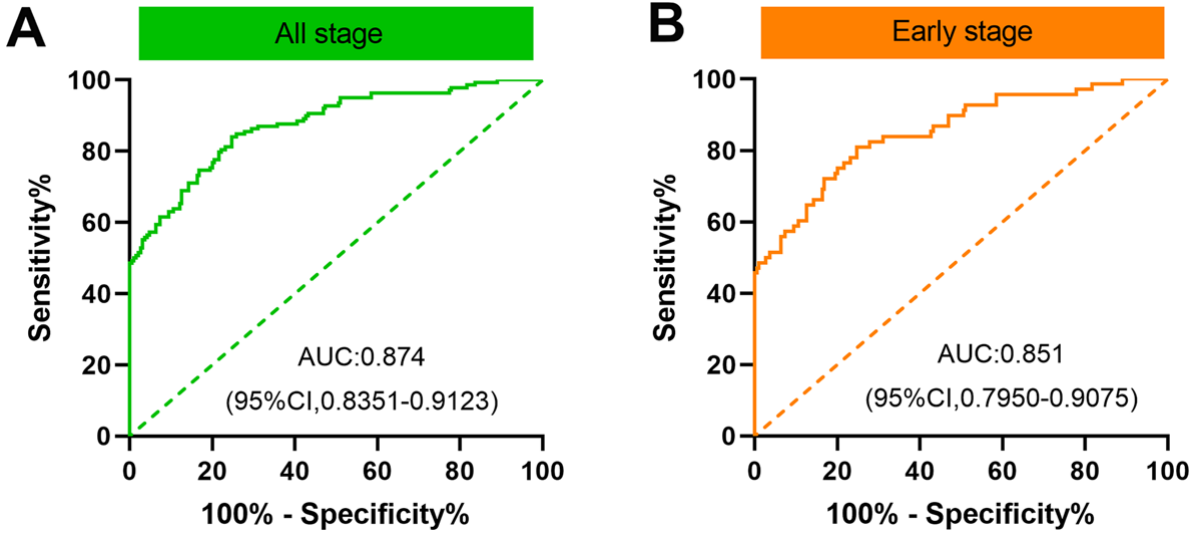
ROC curve analysis in the diagnosis of CRC and early-stage CRC. (**A**) ROC curve for IGFBP-1 in all stage CRC versus normal control. (**B**) ROC curve for IGFBP-1 in early stage of CRC versus normal control. (**C**) Two groups versus normal controls group are in different colors. The area under the red line is 0.5, for reference.

To assess the diagnostic value of serum IGFBP-1 for CRC, we performed the ROC analysis to assess the ability of IGFBP-1 to distinguish CRC patients from normal controls. With an optimum diagnostic cutoff of 1258.387 ng/ml, ROC analysis displayed that serum IGFBP-1 achieved an AUC of 0.874 (95% CI 0.835–0.932) for distinguishing CRC from controls CRC (Fig. 3). The specificity and the sensitivity were 90.53% and 63.04%, respectively (Table 1). In addition, with the same cutoff value, IGFBP-1 could discriminate early-stage CRC from normal controls with a slightly lower AUC value of 0.812 (95% CI 0.795–0.908), a specificity of 90.53% and a sensitivity of 58.82% (Fig. 3 and Table 1). For better interpretation on clinical value of serum IGFBP-1, we also analyzed PPV, NPV, PLR and NLR, and the detail results were shown in Table 1. Interestingly, we evaluated the diagnostic performance of IGFBP-1 in CEA-negative, CA 19-9-negative CRC/early CRC. The results showed that IGFBP-1 also showed high diagnostic efficacy in CEA/CA19-9 negative CRC, and similar results were also obtained in CEA/CA19-9 negative early CRC, as shown in Table 2.

**Table 1:** Evaluation of the detection value of IGFBP-1 in the diagnosis of CRC.

	AUC (95%CI)	Sensitivity (95%CI)	Specificity (95%CI)	PPV (%)	NPV (%)	PLR	NLR
CRC (all stages) versus control	0.874 (0.835–0.912)	63.04% (54.37%–70.99%)	90.53% (85.22%–94.13%)	82.86	77.13	6.655	0.408
Early-stage CRC versus control	0.812 (0.795–0.908)	58.82% (46.24%–70.41%)	90.53% (85.22%–94.13%)	68.97	86.00	6.209	0.455

**Table 2:** Evaluation of the detection value of IGFBP-1 in the diagnosis of CEA/CA19-9-negative early CRC and CRC.

	AUC (95%CI)	Sensitivity (95%CI)	Specificity (95%CI)	PPV (%)	NPV (%)	PLR	NLR
CEA negative Early CRC versus control	0.839 (0.760–0.918)	56.25% (41.28%–70.23%)	84.44% (69.94%–93.01%)	79.41	64.41	3.616	0.518
CA199 negative-Early-stage CRC versus Control	0.836 (0.758–0.914)	58.49% (44.18%–71.5%)	84.44% (69.94%–93.01%)	81.58	63.33	3.760	0.492
CEA negative CRC (all stages) versus control	0.842 (0.774–0.910)	60.00% (48.42%–70.61%)	84.44% (69.94%–93.01%)	87.23	54.29	3.857	0.474
CA199 negative- CRC (all stages) versus control	0.853 (0.791–0.915)	64.84% (54.05%–74.36%)	84.44% (69.94%–93.01%)	89.40	54.29	4.168	0.416

### Correlation between serum levels of IGFBP-1 and clinical data in CRC

We evaluated the relationship between serum IGFBP-1 and clinicopathological characteristics of CRC patients by comparing serum IGFBP-1 positive rate in all 138 CRC patients. We defined that the positive level of serum IGFBP-1 in CRC patients was higher than 1258.387 ng/mL. As shown in Supplementary Tables S3, there were no statistically significant associations between the positive rates of serum IGFBP-1 and depth of tumor invasion, lymph node metastasis, distant metastasis, and early-stage or advanced-stage of CRC, but high IGFBP-1 levels were associated with age (*P* < 0.05) and gender (*P* < 0.05).

### Comparation of the diagnostic capacity of serum IGFBP-1 with CEA and CA19-9

We found that serum CEA and CA19-9 levels were detected in 126 CRC patients and 45 normal controls, according to the medical records and physical examination data, of which the diagnostic values were used to compare with serum IGFBP-1. The two/three-biomarker panel was established employing a logistical regression model with the predicted probability. As shown in Supplementary Fig. S2A,B and Table 3, the diagnostic efficiency of serum IGFBP-1 was significantly higher than those for CEA, CA19-9 or CEA+CA19-9 for both all-stage CRC and early-stage CRC. More importantly, when compared with IGFBP-1 alone, ROC analysis showed that the combination of IGFBP-1 and CEA, IGFBP-1 and CA19-9 or the three-biomarker panel (IGFBP-1+CEA+CA19-9) had a minute improvement of AUC to distinguish all stage CRC patients from controls (Supplementary Fig. S2A, Table 3). However, compared with detection of IGFBP-1 alone, IGFBP-1+CEA, IGFBP1+CA19-9 or the three-biomarker panel would reduce the diagnostic sensitivity (56.45% vs 40.32%, 48.40% or 43.55%, respectively, Supplementary Fig. S2B, Table 3).

**Table 3:** Diagnostic performance of different biomarkers in 126 CRC patients and 45 normal controls.

	AUC (95%CI)	Sensitivity (95%CI)	Specificity (95%CI)	PPV (%)	NPV (%)	PLR	NLR
All stages CRC							
CEA	0.618 (0.536–0.699)	39.68% (31.20%–48.81)	91.11% (77.87%–97.11%)	92.59	35.04	4.464	0.662
CA19-9	0.639 (0.544–0.725)	27.78% (20.35%–36.58%)	97.78% (86.77%–99.88%)	97.22	32.59	12.500	0.739
CEA+CA19-9	0.692 (0.614–0.770)	44.45% (35.68%–53.55%)	91.11% (77.87%–97.11%)	93.33	36.94	5.000	0.610
IGFBP-1	0.843 (0.784–0.902)	59.52% (50.40%–68.06%)	91.11% (77.87%–97.11%)	94.94	44.57	6.696	0.444
IGFBP-1+CEA	0.866 (0.813–0.919)	66.67% (57.64%–74.66%)	91.11% (77.87%–97.11%)	95.45	49.40	7.500	0.366
IGFBP-1+CA19-9	0.876 (0.8236–0.9277)	69.84% (60.93%–77.53%)	91.11% (77.87%–97.11%)	95.65	51.90	7.857	0.331
IGFBP-1+CEA+CA19-9	0.878 (0.8283–0.9301)	66.67% (57.64%–74.66%)	91.11% (77.87%–97.11%)	95.45	49.40	7.500	0.366
Early stage							
CEA	0.513 (0.403–0.623)	27.42% (17.22%–40.44%)	91.11% (77.87%–97.11%)	80.95	47.67	3.085	0.797
CA199	0.533 (0.423–0.643)	14.52% (7.25%–26.28%)	97.78% (86.77%–99.88%)	90.00	45.36	6.532	0.874
CEA+CA19-9	0.578 (0.471–0.686)	14.5% (7.25%–26.28%)	91.11% (77.87%–97.11%)	90.00	45.36	6.532	0.874
IGFBP-1	0.821 (0.743–0.899)	56.45% (43.31%–68.79%)	91.11% (77.87%–97.11%)	89.74	60.29	6.351	0.478
IGFBP-1+CEA	0.829 (0.754–0.904)	40.32% (28.30%–53.54%)	91.11% (77.87%–97.11%)	86.21	52.56	4.536	0.655
IGFBP-1+CA19-9	0.835 (0.760–0.910)	48.40% (35.66%–61.32%)	91.11% (77.87%–97.11%)	88.24	56.16	5.444	0.566
IGFBP-1+CEA+CA19-9	0.829 (0.753–0.905)	43.55% (31.21%–56.68%)	91.11% (77.87%–97.11%)	93.10	55.13	9.798	0.591

### Prognostic value of serum IGFBP-1 in CRC

**Figure 4:**
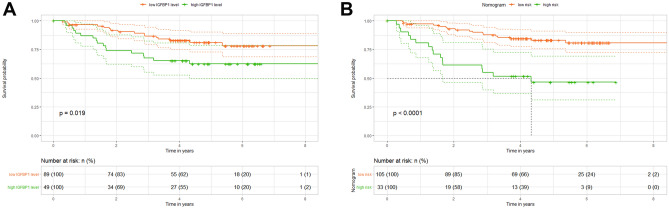
Kaplan–Meier curves for OS of patients with CRC. (**A**) Survival curve for serum IGFBP-1 with CRC patients, Log-rank test was used to evaluate the significant difference. (**B**) Survival curve of risk stratification for OS according to prediction of nomogram. The low risk: Total points ≤ 228.75 for OS, the high risk: Total points > 228.75 for OS. Log-rank test was applied to assess the significant difference. The number of people alive at each time point in the high and low IGFBP-1 groups was showed in “number at risk.”

We investigated whether serum IGFBP-1 could be used to predict prognosis of CRC. The 138 CRC patients recruited in this study were included for prognostic analysis. Using X-tile software, we set 1781.120 ng/mL as the optimal cut-off value to classify high and low expression of IGFBP-1. Univariate Cox analysis showed that age, gender, N stage, M stage, TNM stage and serum IGFBP-1 expression level were associated with prognosis of CRC. Multivariate Cox analysis further demonstrated that M stage (*P* = 0.010, HR = 3.811, 95%CI 1.373–10.580), TNM stage (*P* = 0.007, HR = 3.106, 95%CI 1.363–7.077) and serum IGFBP-1 (*P* = 0.045, HR = 2.043, 95%CI 1.014–4.115) were independent factors to predict the prognosis of CRC (Table 4). According to multivariate Cox proportional hazards regression analysis, the forest map was used to visualize the significant correlation between M stage, TNM stage, IGFBP-1 expression and OS of CRC patients (Supplementary Fig. S3). Furthermore, Kaplan–Meier revealed that the 5-year OS of CRC patients with high serum IGFBP- level was shorter than those with low serum IGFBP-1 level (16% vs 32%), and the difference was statistically significant (*P* = 0.019) (Fig. 4A).

**Table 4:** Cox analysis of OS for CRC.

Variable	Univariate analysis		Multivariate analysis	
	HR (95%CI)	*p*	HR (95%CI)	*p*
Patient age (> 50 vs ≤ 50)	0.684 (1.063–6.953)	0.042		
Patient gender (male vs female)	2.200 (1.046–4.624)	0.038	2.369 (0.908–6.178)	0.078
Smoking behavior (yes vs no)	0.615 (0.216–2.750)	0.362		
Drinking behavior (yes vs no)	0.809 (0.110–5.926)	0.835		
T stage (T3+T4 vs Tis+T1+T2)	2.174 (0.764–6.185)	0.146		
N stage (N1+N2+N3 vs N0)	3.725 (1.678–8.268)	0.001		
M stage (M1 vs M0)	6.118 (2.334–16.037)	0.000	3.811 (1.373–10.580)	0.010
TNM stage (Advanced stage vs Early stage)	3.725 (1.678–8.268)	0.001	3.106 (1.363–7.077)	0.007
histological type (Adenocarcinoma vs Mucinous and intraepithelial carcinoma)	0.909 (0.124–6.669)	0.926		
vascular invasion (yes vs no)	0.828 (0.253–2.714)	0.755		
IGFBP-1 (lower vs higher)	2.118 (1.070–4.194)	0.031	2.043 (1.014–4.115)	0.045

### Nomogram for OS of CRC

**Figure 5:**
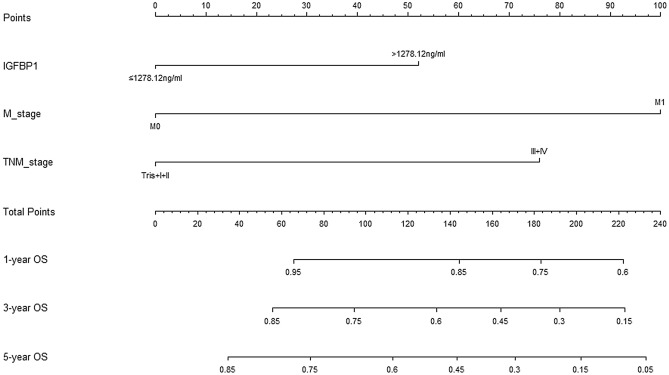
Nomogram based on M stage, TNM stage and serum IGFBP-1 in the prediction of 1-, 3-, and 5-year OS probability in CRC patients. The total points projected on the bottom scales show the probability of 1-, 3-, and 5-year survival, A vertical line could be drawn from the “Total Points” to the axis to mark 1-, 3-, and 5-year OS, and a larger “Total Points” score would represent a worse OS for patients.

**Figure 6:**
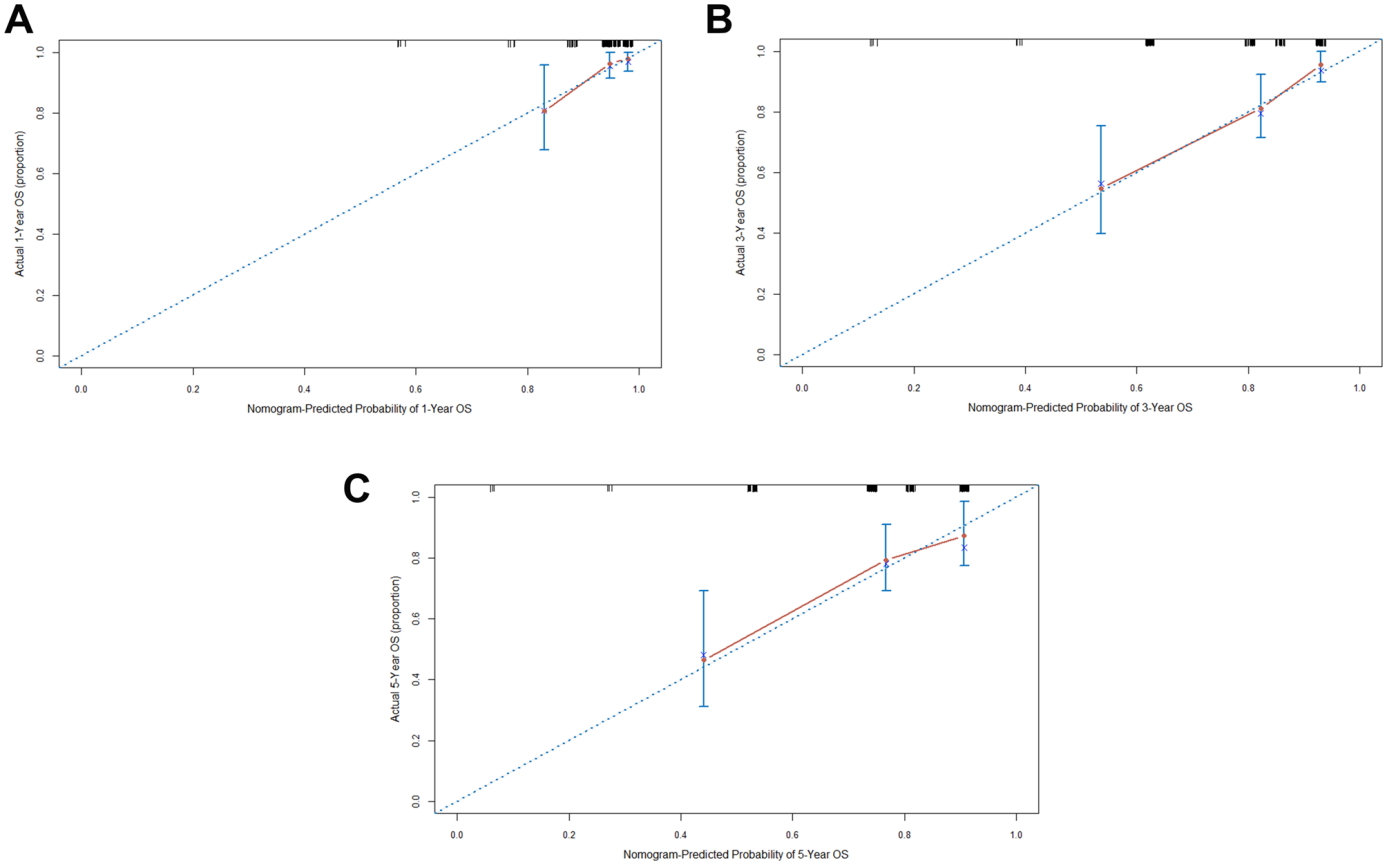
The calibration plots (**A**–**C**) are used to estimate predictive capacity of the nomogram for 1-, 3-, and 5-year OS probability. X-axis was the probability of 1-, 3- or 5-year OS predicted by nomogram. Y-axis was the actual OS of the patients included in the study.

**Figure 7:**
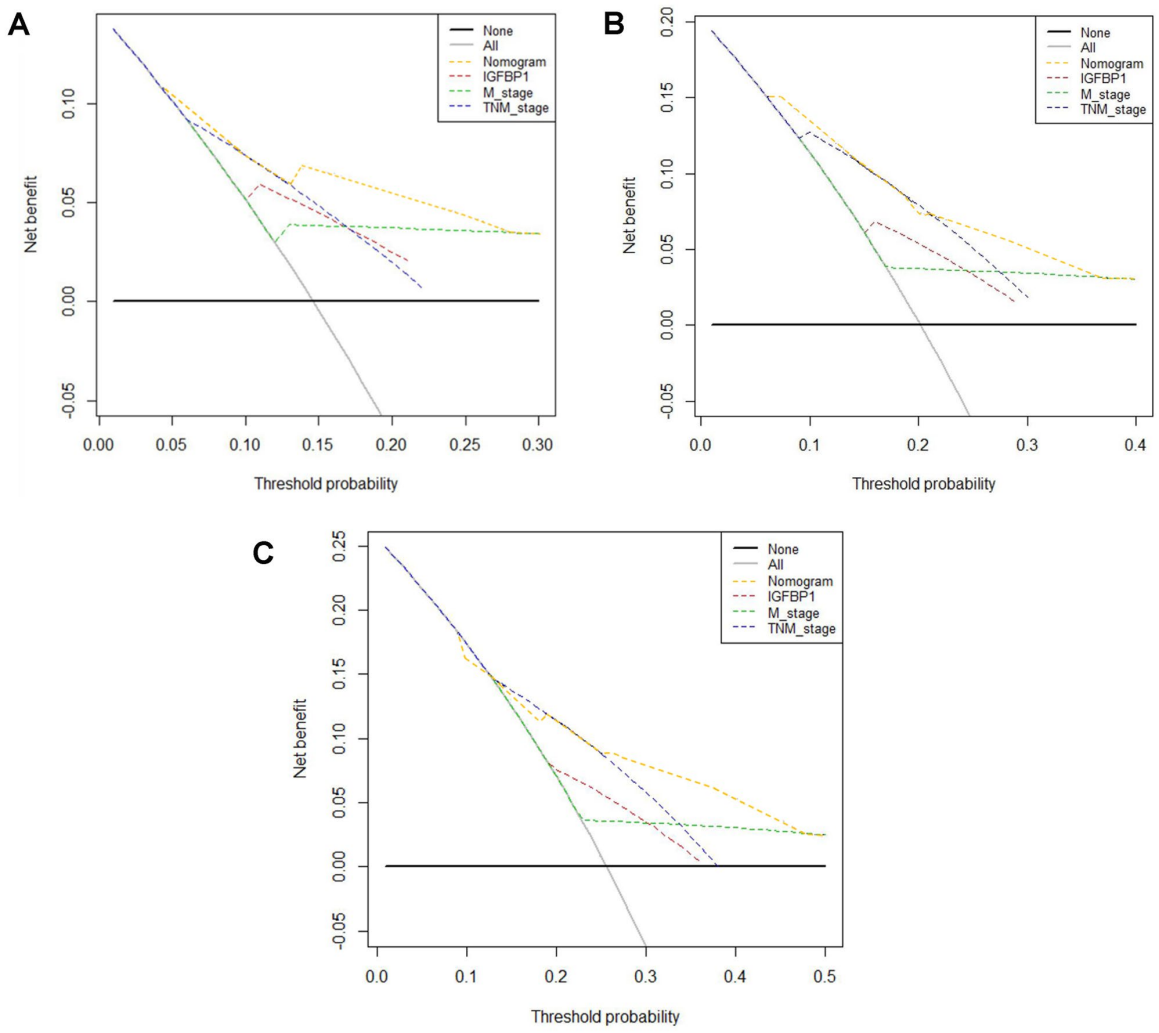
Decision curve analysis the predictive accuracy of M stage, TNM stage, IGFBP-1 and nomogram in CRC patients. The decision curve of 1- (**A**), 3- (**B**), and 5- (**C**) year OS. The y-axis represents the net benefit, which is calculated by summing the benefits (true positive results) and subtracting the harms (false positive results). The horizontal line represents the assumption that no deaths happen.

In order to better evaluate the prognosis of patients with CRC, the independent prognostic factors of M stage, TNM stage, and serum IGFBP-1 were used to establish a nomogram to forecast 1-, 3- and 5-years OS probability prediction (Fig. 5). From the nomogram, each prognostic factor was assigned a number of risk points, which was obtained by drawing a straight line directly upward to the “points” axis from the corresponding value of the prognostic factor. These points were added together to obtain “total points”. The relationship between M stage, TNM stage, serum IGFBP-1 and OS can be visually shown in the nomogram (Fig. 5). The calibration plots were used to evaluate predictive capacity of the nomogram for 1-, 3-, and 5-year OS (Fig. 6). The results showed that the nomogram had a good prediction accuracy for OS. Akaike information criterion (AIC), bayesian information criterion (BIC) and C-index were used to evaluate the goodness-of-fit and discriminative ability of the nomogram. As shown in Table 5, the AIC and BIC of the nomogram were lower than those of TNM stage (291.994 vs 305.737; 296.483 vs 307.234, respectively), suggesting that the nomogram had a higher goodness-of-fit for predicting OS. The C-index for the nomogram was 0.714 (95%CI 0.623–0.804), which was higher than that of TNM staging (0.651, 95%CI 0.575–0.727, *P* = 0.043). Time-dependent C-index analysis also showed that the nomogram showed a good prognostic accuracy for OS when compared with other single prognostic factors (Supplementary Fig. S4). Moreover, the DCA (Fig. 7) showed that the nomogram has higher overall net benefit than the traditional TNM stage systems alone in the majority of the range of threshold probabilities. Taken together, these results demonstrated that the nomogram had a better performance to predict OS of CRC patients when compared to the traditional TNM stage systems.

**Table 5:** The C-index of IGFBP-1, M stage, TNM stage and nomogram for prediction of OS in CRC.

Factors	C-index (95% CI)	*P*-value	AIC	BIC
IGFBP-1	0.603 (0.518–0.688)		305.737	307.234
M stage	0.570 (0.507–0.632)		301.243	302.744
TNM stage	0.651 (0.575–0.727)		297.916	299.412
Nomogram	0.714 (0.623–0.804)		291.994	296.483
Nomogram versus IGFBP-1		0.008		
Nomogram versus M stage		0.000		
Nomogram versus TNM stage		0.043		

*AIC* Akaike information criterion, *BIC* Bayesian information criterion.

### Risk stratification based on the nomogram

In order to determine whether the CRC patients could be effectively divided into two proposed risk groups based on the nomogram and OS, we calculated each patient’s total point, and used the X-tile program to obtain the optimal cut-off value (1781.16 ng/ml) to subdivide patients into low- and high-risk subgroups. Kaplan–Meier analysis and log-rank test showed that the high-risk group had shorter OS than those in the group of low-risk (39% vs 66%, *P* < 0.0001), and the median OS of CRC patients was less than 5 years (Fig. 4B). This stratification demonstrated that the nomogram could effectively divide those patients into the 2 risk subgroups with significant differences in OS.

## Discussion

It is well known that endoscopy examination could help identify early-stage CRC^8,34^, but the invasiveness of colonoscopy limits its widely apply as a screening tool in a large number of asymptomatic populations^35^. Besides, the effectiveness of colonoscopy as a screening tool is closely related to the adequate detection and removal of colonic polyps, and the detection rate of colonoscopy is operator dependent **in** some extent^9^. In addition, some other noninvasive screening tests of CRC including stool blood tests, serum CEA, CA19-9 and blood septin9 gene tests were used to reduce the incidence and mortality of CRC^22,36–38^. Nevertheless, because of the low sensitivity or high cost, none of these methods has been established as a recognized early screening tool^23,39–41^. Therefore, it is urgent to find new methods with high sensitivity and specificity for the early diagnosis of CRC. In recent years, serum protein biomarkers are considered to be the most promising detection methods for population studies and routine clinical work^17,42,43^, because cancer phenotypic features seem most likely to be a direct reflection of changes in protein metabolism and function, which are also the targets of most anticancer drugs in clinical practice^44^, and these serum samples are noninvasive and easily accessible. Furthermore, a growing interest in the clinically potential use of circulating IGFBPs as diagnostic or prognostic biomarkers in cancers^45^. Our previous studies demonstrated that serum IGFBPs showed good diagnostic or prognostic performance in upper gastrointestinal cancer, including IGFBP-1^31,46–48^. In this study, we found that blood IGFBP-1 mRNA is increased in GEO CRC patient dataset. In addition, in consideration of IGFBP-1 is a secreted protein, we carried out ELISA experiment to detect the levels of serum IGFBP-1 protein, and found that serum IGFBP-1 expression was significantly increased in CRC, compared to normal controls (*P* < 0.0001). Meanwhile, serum IGFBP-1 showed a good diagnostic value in all stages of CRC with an AUC of 0.874, a specificity of 90.53% and a sensitivity of 63.04%.

In terms of cancer screening, one of the most important attributes of a biomarker should be able to identify early cancers. For early-stage CRC, a certain diagnostic accuracy of serum IGFBP-1 could be observed (AUC 0.812, specificity 90.53% and sensitivity 58.82%). It is reported that CEA or CA-199 did not provide sufficient sensitivity and reliability for the early detection of CRC^21,23,49^. Similarly, our results also suggested that CEA and CA199 showed low diagnostic performance for early-stage CRC. Compared with CEA, CA19-9 or the two-biomarker combined panel (CEA+CA19-9), serum IGFBP-1 has higher sensitivity and AUC value to identify early-stage CRC (Table 3). Therefore, we believe that serum IGFBP-1 protein detection might be helpful for early diagnosis of CRC. Additionally, the combination of IGFBP-1 and CEA, IGFBP-1 and CA19-9, or the three-biomarker combined panel may further increase the diagnostic efficacy, especially with increased sensitivity, but there is no such effect for early-stage patients (Table 3).

Although genetics plays an important role in risk stratification, the risk of developing CRC is also influenced by acquired factors, including age, race, male gender, and dietary habits^40^. Age is one of the risk factors that has been taken into account in most screening recommendations^50^. CRC death rates rose 1.2% per year in people younger than 50 years and 0.6% per year in those aged 50 to 54 years from 2005 to 2019^51^. Whereas a recent study showed similar prevalence at ages 45 to 49 years and 50 to 54 years, which provide clinically useful evidence for optimizing the age in CRC prevention and screening^52^. In our study, we found that the positive rate of serum IGFBP-1 was closely related to patient age.

On one hand, IGFBP-1 is expressed in fetal liver and postpartum tissue, mainly in secretory endometrium, decidua gravidarum and liver. Pathologically, the expression of IGFBP-1 is elevated type 2 diabetes and some cancer patients^25^. Moreover, increasing studies indicate the expression and prognostic value of IGFBP-1 in serum/tissue of patients with cancer remains equivocal and even controversial^53–58^. These results indicated that IGFBP-1 might have different expression pattern and prognostic value in different carcinomas, and the prognostic potential of serum IGFBP-1 in cancers should be further evaluated. So far, there is little literature involving the expression and the diagnostic or prognostic value of serum IGFBP-1 in CRC. Previously, most studies have focused on evaluating the relationship between circulating levels of IGFBP-1 and the risk of CRC, and the results seem to be contradictory. It is reported that higher plasma/serum IGFBP-1 levels were associated with a decreased risk of CRC^59,60^. Analogously, Vidal et al. revealed that lower concentrations of the plasma IGFBP-1 were associated with an increased risk of CRC, whereas higher levels of IGFBP-1 were related with a decreased risk of CRC in men only^61^. On the contrary, Wei et al. found that low blood IGFBP-1 expression was not obviously related with the increased risk of CRC^62^. Palmqvist et al. also reported similar results^63^. On the other hand, many in vitro and in vivo studies support the dual role of IGFBP-1 in tumor proliferation, migration, invasion, and adhesion through both IGF-dependent and IGF-independent molecular mechanisms, suggesting that the effects of IGFBP-1 are cell-specific and dependent on the type of target cells^25,30,56^. Moreover, as described in the literature, IGFBP-1 not only inhibited the invasion and migration of CRC cells SW480 and SW620, but also promoted CRC liver metastasis in mice transplanted with SW480 cells^64^. These findings suggest that IGFBP-1 may have a dual function, playing both positive and negative roles in the progression and metastasis of CRC. Liver metastasis (CLM) occurred only in mice injected with IGFBP-1 overexpressed SW480 cells, but CLM was not found in igfbp1 overexpressed SW620 cells, which may be related to the persistent expression of β-catenin, vimentin, and ZO-1 in IGFBP-1 overexpressed SW480 cells^64^. These results suggest that the role of IGFBP-1 in CRC is complex, but the specific mechanisms have not been clearly elucidated. Because there have been few studies on the role of IGFBP-1 in the development of colorectal cancer and the molecular mechanisms of action. Therefore, in future study, it is important to further explore its biological function in CRC. In our study, we identified that patients with higher serum IGFBP-1 expression had a worse OS. In addition, risk stratification demonstrated that the nomogram could effectively divide those patients into the high- and low-risk subgroups, and patients in the high-risk group had shorter OS. On the other hand, prognostic assessment of CRC patients remains a great clinical challenge. TNM stage system is the primary basis in estimating the prognosis of CRC^65^. However, TNM stage is based fully on the anatomical range of the disease, and may be affected by pathological assessment and tumor heterogeneity, which has limitations for survival analysis of CRC patients^66^. Here, we identified that serum IGFBP-1 was an independent prognostic factor. The nomogram based on serum IGFBP-1, M stage and TNM stage was established, of which the C-index was better than that of the TNM stage alone. A similar result was also observed in time-dependent C-index curve analysis. In addition, the decision curve analysis for 1-, 3-, and 5-year OS showed that the nomogram seemed to have higher overall benefit than TNM stage systems alone. These findings indicated that the nomogram seems to be more accurate in predicting OS than the traditional TNM stage system. In the study, age and lymph node metastasis were excluded after multivariate analysis, possibly due to sample size and clinical characteristics of patients. Therefore, it is necessary to verify whether age and lymph node metastasis are independent prognostic predictors with further large samples in the future.

Several limitations of this study should be also taken into account: in our study, all patients were recruited from individuals with known cancers, and most were diagnosed based on the clinical symptoms of the disease. Nevertheless, the diagnostic sensitivity of IGFBP-1 may be different in individuals with asymptomatic disease or precancerous lesions, which needs to be further verified. Furthermore, these results were assessed in a single-institution study, which may lead to bias. Moreover, this study did not analyze the levels of serum IGFBP1 in patients of benign intestinal disease, which should be used as disease controls to assess the efficacy of serum IGFBP-1. Future studies need to address the role of IGFBP-1 in benign intestinal disease. The number of subjects in the control and CRC groups to compare the diagnostic value between IGFBP-1 and the classic clinical tumor markers (CEA and CA19-9) was not balanced, which may reduce statistical power. Whether serum IGFBP-1 had improved performance in the early diagnosis of CRC still needs further validation. Finally, there is a selection bias in the male-to-female ratio within the CRC group and control group. Future studies also need to verify whether the male-to-female ratio difference may influence the results. Therefore, in future study, a study with multicenter and large-scale samples should be completed to further verify the diagnostic and prognostic value of IGFBP-1 in CRC, and need to further evaluate the role of IGFBP1 in benign bowel disease.

In conclusion, our findings offer a convenient and non-invasive for early diagnosis and prediction of outcomes for patients with CRC, as the blood-based IGFBP-1 are easy to obtain from routine admission laboratory tests. Meanwhile, we believe that serum IGFBP-1 detection is not meant to replace endoscopy or traditional prognostic assessment strategy, but contributes to identifying patients who may have CRC at an early stage. Furthermore, our study demonstrated that serum IGFBP-1 is an independent prognostic risk factor for CRC patients, and the construction of nomogram model containing serum IGFBP-1 might improve the accuracy of prognosis prediction.

### Supplementary Information

Supplementary Information.

## Data Availability

Data included in article/supplementary material/referenced in article.

## References

[1] Sung H (2021). Global cancer statistics 2020: GLOBOCAN estimates of incidence and mortality worldwide for 36 cancers in 185 countries. CA Cancer J. Clin..

[2] Arnold M (2017). Global patterns and trends in colorectal cancer incidence and mortality. Gut.

[3] Li N (2021). Incidence, mortality, survival, risk factor and screening of colorectal cancer: A comparison among China, Europe, and northern America. Cancer Lett..

[4] Liu S (2015). Incidence and mortality of colorectal cancer in China, 2011. Chin. J. Cancer Res..

[5] Siegel RL (2017). Colorectal cancer statistics, 2017. CA Cancer J. Clin..

[6] Levin TR (2011). Organized colorectal cancer screening in integrated health care systems. Epidemiol. Rev..

[7] Dekker E, Rex DK (2018). Advances in CRC prevention: Screening and surveillance. Gastroenterology.

[8] Berg AO (2002). Screening for colorectal cancer: Recommendation and rationale. Ann. Intern. Med..

[9] Maida M (2017). Screening of colorectal cancer: Present and future. Expert Rev. Anticancer Ther..

[10] Levin B (2008). Screening and surveillance for the early detection of colorectal cancer and adenomatous polyps, 2008: A joint guideline from the American cancer society, the US multi-society task force on colorectal cancer, and the American college of radiology. Gastroenterology.

[11] Coppedè F (2014). Genetic and epigenetic biomarkers for diagnosis, prognosis and treatment of colorectal cancer. World J. Gastroenterol..

[12] Hardcastle JD (1996). Randomised controlled trial of faecal-occult-blood screening for colorectal cancer. Lancet.

[13] von Karsa L (2013). European guidelines for quality assurance in colorectal cancer screening and diagnosis: Overview and introduction to the full supplement publication. Endoscopy.

[14] Gellad ZF (2011). Longitudinal adherence to fecal occult blood testing impacts colorectal cancer screening quality. Am. J. Gastroenterol..

[15] Young GP (2015). Advances in fecal occult blood tests: The FIT revolution. Dig. Dis. Sci..

[16] Imperiale TF (2014). Multitarget stool DNA testing for colorectal-cancer screening. N. Engl. .J Med..

[17] Yörüker EE, Holdenrieder S, Gezer U (2016). Blood-based biomarkers for diagnosis, prognosis and treatment of colorectal cancer. Clin. Chim. Acta..

[18] Binefa G (2014). Colorectal cancer: From prevention to personalized medicine. World J. Gastroenterol..

[19] Jimenez CR (2010). Proteomics of colorectal cancer: overview of discovery studies and identification of commonly identified cancer-associated proteins and candidate CRC serum markers. J. Proteom..

[20] Tanaka T (2010). Biomarkers for colorectal cancer. Int. J. Mol. Sci..

[21] Łukaszewicz-Zając M, Mroczko B (2021). Circulating biomarkers of colorectal cancer (CRC)—Their utility in diagnosis and prognosis. J. Clin. Med..

[22] Hundt S, Haug U, Brenner H (2007). Blood markers for early detection of colorectal cancer: A systematic review. Cancer Epidemiol. Biomark. Prev..

[23] Nikolaou S (2018). Systematic review of blood diagnostic markers in colorectal cancer. Tech. Coloproctol..

[24] Newton KF, Newman W, Hill J (2012). Review of biomarkers in colorectal cancer. Colorectal Dis..

[25] Hoeflich A, Russo VC (2015). Physiology and pathophysiology of IGFBP-1 and IGFBP-2—Consensus and dissent on metabolic control and malignant potential. Best Pract. Res. Clin. Endocrinol. Metab..

[26] Gleeson LM (2001). Insulin-like growth factor-binding protein 1 stimulates human trophoblast migration by signaling through alpha 5 beta 1 integrin via mitogen-activated protein Kinase pathway. J. Clin. Endocrinol. Metab..

[27] Chesik D (2010). Insulin-like growth factor binding protein-1 activates integrin-mediated intracellular signaling and migration in oligodendrocytes. J. Neurochem..

[28] Ammoun S (2012). Insulin-like growth factor-binding protein-1 (IGFBP-1) regulates human schwannoma proliferation, adhesion and survival. Oncogene.

[29] Hwang DL (2003). Elevated insulin, proinsulin and insulin-like growth factor-binding protein-1 in liver disease. Growth Horm. IGF Res..

[30] Lin YW (2021). IGFBP-1 in cancer: Expression, molecular mechanisms, and potential clinical implications. Am. J. Transl. Res..

[31] Xu YW (2020). Serum IGFBP-1 as a potential biomarker for diagnosis of early-stage upper gastrointestinal tumour. EBioMedicine.

[32] Amin MB (2017). The eighth edition AJCC cancer staging manual: Continuing to build a bridge from a population-based to a more "personalized" approach to cancer staging. CA Cancer J. Clin..

[33] Boyle P (2011). Clinical validation of an autoantibody test for lung cancer. Ann. Oncol..

[34] Lieberman DA (2012). Guidelines for colonoscopy surveillance after screening and polypectomy: A consensus update by the US multi-society task force on colorectal cancer. Gastroenterology.

[35] Cooper GS, Kou TD, Rex DK (2013). Complications following colonoscopy with anesthesia assistance: A population-based analysis. JAMA Intern. Med..

[36] Mandel JS (1993). Reducing mortality from colorectal cancer by screening for fecal occult blood. Minnesota colon cancer control study. N. Engl. J. Med..

[37] deVos T (2009). Circulating methylated SEPT9 DNA in plasma is a biomarker for colorectal cancer. Clin. Chem..

[38] Rawson JB, Bapat B (2012). Epigenetic biomarkers in colorectal cancer diagnostics. Expert Rev. Mol. Diagn..

[39] Church TR (2014). Prospective evaluation of methylated SEPT9 in plasma for detection of asymptomatic colorectal cancer. Gut.

[40] Rex DK (2017). Colorectal cancer screening: Recommendations for physicians and patients from the U.S. Multi-society task force on colorectal cancer. Am. J. Gastroenterol..

[41] Hariharan R, Jenkins M (2020). Utility of the methylated SEPT9 test for the early detection of colorectal cancer: A systematic review and meta-analysis of diagnostic test accuracy. BMJ Open Gastroenterol..

[42] Borrebaeck CA (2017). Precision diagnostics: Moving towards protein biomarker signatures of clinical utility in cancer. Nat. Rev. Cancer.

[43] Chu LY (2020). The diagnostic value of serum L1CAM in patients with colorectal cancer. Technol. Cancer Res. Treat..

[44] Belczacka I (2019). Proteomics biomarkers for solid tumors: Current status and future prospects. Mass Spectrom. Rev..

[45] Baxter RC (2014). IGF binding proteins in cancer: mechanistic and clinical insights. Nat. Rev. Cancer.

[46] Huang X (2019). The diagnostic value of serum IGFBP7 in patients with esophageal squamous cell carcinoma. J. Cancer.

[47] Liu CT (2020). Serum insulin-like growth factor binding protein 7 as a potential biomarker in the diagnosis and prognosis of esophagogastric junction adenocarcinoma. Gut Liver.

[48] Luo Y (2022). Serum insulin-like growth factor binding protein-3 as a potential biomarker for diagnosis and prognosis of oesophageal squamous cell carcinoma. Ann. Med..

[49] Lakemeyer L (2021). Diagnostic and prognostic value of CEA and CA19-9 in colorectal cancer. Diseases.

[50] Burnett-Hartman AN (2021). An update on the epidemiology, molecular characterization, diagnosis, and screening strategies for early-onset colorectal cancer. Gastroenterology.

[51] Siegel RL (2022). Cancer statistics, 2022. CA Cancer J. Clin..

[52] Butterly LF (2021). Colonoscopy outcomes in average-risk screening equivalent young adults: Data from the new hampshire colonoscopy registry. Am. J. Gastroenterol..

[53] Sato Y (2019). IGFBP1 is a predictive factor for haematogenous metastasis in patients with gastric cancer. Anticancer Res..

[54] Feng X (2017). Higher IGFBP-1 to IGF-1 serum ratio predicts unfavourable survival in patients with nasopharyngeal carcinoma. BMC Cancer.

[55] Abou-Alfa GK (2014). A phase II study of cixutumumab (IMC-A12, NSC742460) in advanced hepatocellular carcinoma. J. Hepatol..

[56] Dai B (2014). Insulin-like growth factor binding protein-1 inhibits cancer cell invasion and is associated with poor prognosis in hepatocellular carcinoma. Int. J. Clin. Exp. Pathol..

[57] Goodwin PJ (2002). Insulin-like growth factor binding proteins 1 and 3 and breast cancer outcomes. Breast Cancer Res. Treat..

[58] Schernhammer ES (2005). Circulating levels of insulin-like growth factors, their binding proteins, and breast cancer risk. Cancer Epidemiol. Biomark. Prev..

[59] Le Marchand L (2010). Associations of plasma C-peptide and IGFBP-1 levels with risk of colorectal adenoma in a multiethnic population. Cancer Epidemiol. Biomark. Prev..

[60] Jenab M (2007). Serum C-peptide, IGFBP-1 and IGFBP-2 and risk of colon and rectal cancers in the European prospective investigation into cancer and nutrition. Int. J. Cancer.

[61] Vidal AC (2012). Elevated C-peptide and insulin predict increased risk of colorectal adenomas in normal mucosa. BMC Cancer.

[62] Wei EK (2006). C-peptide, insulin-like growth factor binding protein-1, glycosylated hemoglobin, and the risk of distal colorectal adenoma in women. Cancer Epidemiol. Biomark. Prev..

[63] Palmqvist R (2003). Plasma insulin, IGF-binding proteins-1 and -2 and risk of colorectal cancer: A prospective study in northern Sweden. Int. J. Cancer.

[64] Kim JC (2016). Complex behavior of ALDH1A1 and IGFBP1 in liver metastasis from a colorectal cancer. PLoS One.

[65] Yao H, Wu H, Liu Y (2017). Improvement of prognostic and predictive network of colorectal cancer based upon the 8th edition of AJCC colorectal cancer staging system. Zhonghua Wei Chang Wai Ke Za Zhi.

[66] Shia J (2012). TNM staging of colorectal carcinoma: issues and caveats. Semin. Diagn. Pathol..

