# Supplementary material for: Serum IGFBP-1 as a promising diagnostic and prognostic biomarker for colorectal cancer
Source: Sci Rep. 2024 Jan 22;14:1839. doi: 10.1038/s41598-024-52220-2 (PMC10800337; doi:10.1038/s41598-024-52220-2)
Supplement: Supplementary file 1 — Supplementary Information. [file 41598_2024_52220_MOESM1_ESM.zip › Supplementary data/Supplememtary Tables.docx]

| **Supplementary Table S1.** Participant information and clinicopathological characteristics. | | |
| --- | --- | --- |
| Group | CRC patients(n=138) | Normal controls(n=190) |
| Gender |  |  |
| Male | 78 | 147 |
| Female | 60 | 43 |
| Age(years) |  |  |
| Mean±SD | 58±11 | 56±10 |
| Range | 26-82 | 40-80 |
| Smoking behavior |  |  |
| Yes | 26 |  |
| No | 112 |  |
| Drinking behavior |  |  |
| Yes | 7 |  |
| No | 131 |  |
| T stage |  |  |
| Tis | 1 |  |
| T1 | 5 |  |
| T2 | 22 |  |
| T3 | 48 |  |
| T4 | 62 |  |
| N stage |  |  |
| N0 | 68 |  |
| N1 | 42 |  |
| N2 | 28 |  |
| N3 | 0 |  |
| M stage |  |  |
| M0 | 130 |  |
| M1 | 8 |  |
| TNM stage |  |  |
| 0 | 1 |  |
| I | 21 |  |
| II | 46 |  |
| III | 62 |  |
| IV | 8 |  |

| **Supplementary Table S2.** Comparison of serum IGFBP-1 level between three groups. | | | | |
| --- | --- | --- | --- | --- |
|  | n | Mean ± SD | *P*^*^ value | Positive (%, 95%CI) |
| CRC | 138 | 1569.455±770.209 | <0.0001 | 87(63.0,1.549-1.712) |
| Early-stage CRC(0+I+II) | 68 | 1512.222±818.971 | <0.0001 | 40(58.8,1.468-1.708) |
| Normal controls | 190 | 719.991 ±379.340 |  | 18(9.5,1.053-1.137) |
| *compared with normal controls. CRC, colorectal cancer. | | | | |

| **Supplementary Table S3.** Relationship between positive rates of IGFBP-1 and clinicopathologic features in CRC patients. | | | | |
| --- | --- | --- | --- | --- |
| Group | n | Positive (%) | **χ^2^** | *p* |
| Patient age |  |  |  |  |
| ≤50 | 40 | 20(50.0%) | 4.113 | 0.043 |
| >50 | 98 | 67(68.4%) |  |  |
| Patient gender |  |  |  |  |
| Male | 78 | 55(70.5%) | 4.296 | 0.038 |
| Female | 60 | 32(53.3%) |  |  |
| Smoking behavior |  |  |  |  |
| Yes | 26 | 20(76.9%) | 3.768 | 0.052 |
| No | 112 | 67(59.8%) |  |  |
| Drinking behavior |  |  |  |  |
| Yes | 6 | 6(100%) | 3.677 | 0.055 |
| No | 132 | 81(61.4%) |  |  |
| T stage |  |  |  |  |
| Tis+T1+T2 | 28 | 15(53.5%) | 1.353 | 0.245 |
| T3+T4 | 110 | 72(65.5%) |  |  |
| N stage |  |  |  |  |
| N0 | 68 | 40(58.8%) | 1.025 | 0.311 |
| N1+N2+N3 | 70 | 47(67.1%) |  |  |
| M stage |  |  |  |  |
| M0 | 130 | 80(61.5%) | 2.180 | 0.140 |
| M1 | 8 | 7(87.5%) |  |  |
| TNM stage |  |  |  |  |
| Early (0+I+II) | 68 | 40(58.8%) | 1.025 | 0.311 |
| Advanced (III+IV) | 70 | 47(67.1%) |  |  |
| NOTE: Statistical significance was determined by means of Chi-squared test. CRC, colorectal cancer. | | | | |
