# Supplementary material for: Serum IGFBP-1 as a promising diagnostic and prognostic biomarker for colorectal cancer
Source: Sci Rep. 2024 Jan 22;14:1839. doi: 10.1038/s41598-024-52220-2 (PMC10800337; doi:10.1038/s41598-024-52220-2)
Supplement: Supplementary file 1 — Supplementary Information. [file 41598_2024_52220_MOESM1_ESM.zip › Supplementary data/supplementary figures/Supplementary figure legends.docx]

**Supplementary Figure S1. The expression of blood IGFBP-1 mRNA in GEO CRC expression profile dataset.** A. Scatter plots of blood IGFBP-1 mRNA from CRC patients and normal controls. Black horizontal lines are means, and error bars are SEs. B. Box plot illustrates median levels and interquartile ranges and the whiskers show minimum and maximum value of blood IGFBP-1mRNA in normal controls and CRC patients. * represents *P* < 0.05. CRC: colorectal cancer.

**Supplementary Figure S2. The ROC curve analysis the diagnosis of different biomarker in CRC.** Seven groups versus normal controls group are in different colors. A. ROC curve for different biomarkers in discriminating all stage CRC patients from normal controls. B. ROC curve for different biomarkers in distinguishing early-stage CRC patients from normal controls.

**Supplementary Figure S3. Forest plot showed the HR and 95% CI for OS based on the Cox proportional hazards regression analysis in CRC patients.**

HR, hazard ratio; CI, confidence interval

**Supplementary Figure S4. C-index curve to evaluate the predictive accuracy of the nomogram.** C-index curve under the time distribution of 3 years(A) and 5 years (B).
