# Supplementary figures and images for: Serum IGFBP-1 as a promising diagnostic and prognostic biomarker for colorectal cancer
Source: Sci Rep. 2024 Jan 22;14:1839. doi: 10.1038/s41598-024-52220-2 (PMC10800337; doi:10.1038/s41598-024-52220-2)

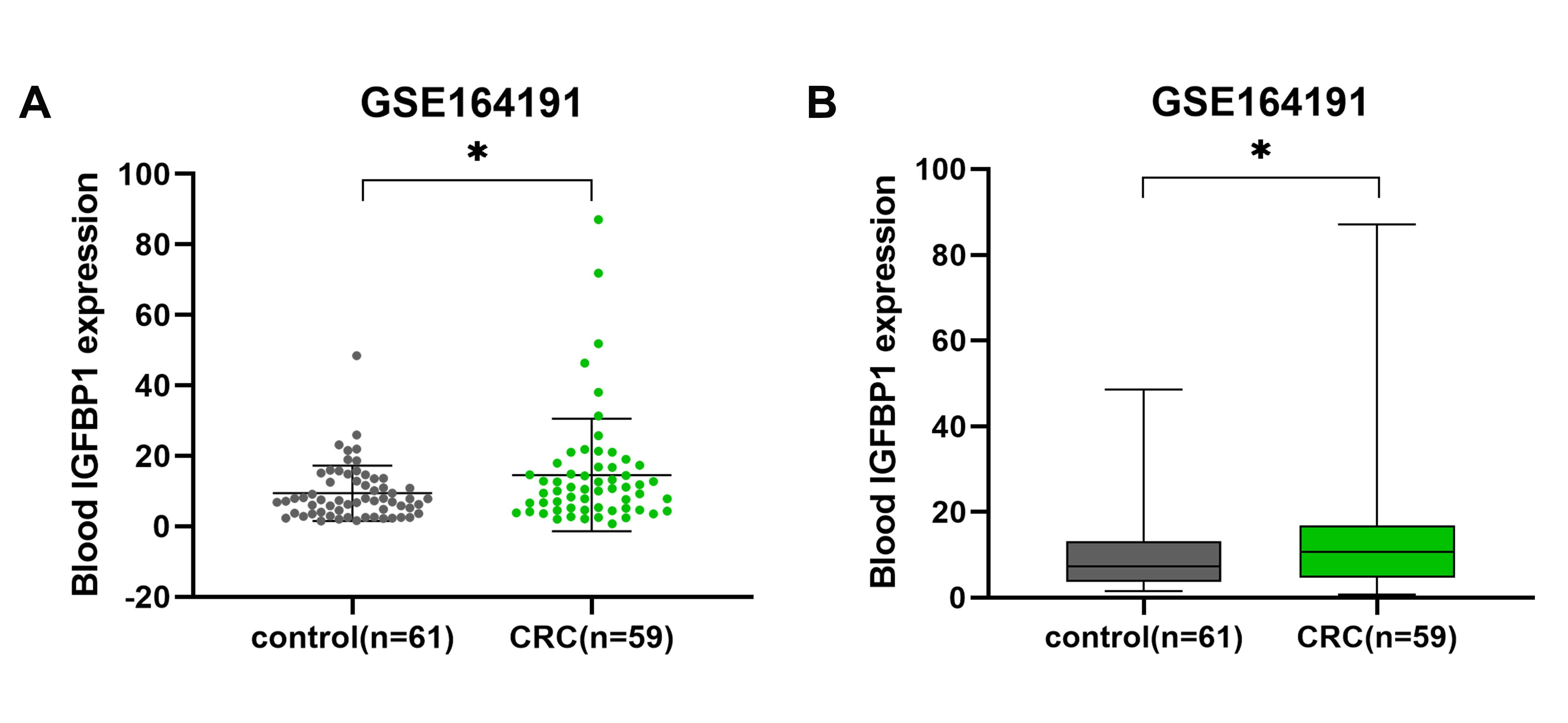

Supplement: Supplementary file 1 — Supplementary Information. [file 41598_2024_52220_MOESM1_ESM.zip › Supplementary data/supplementary figures/Supplementary Figure S1.tif]

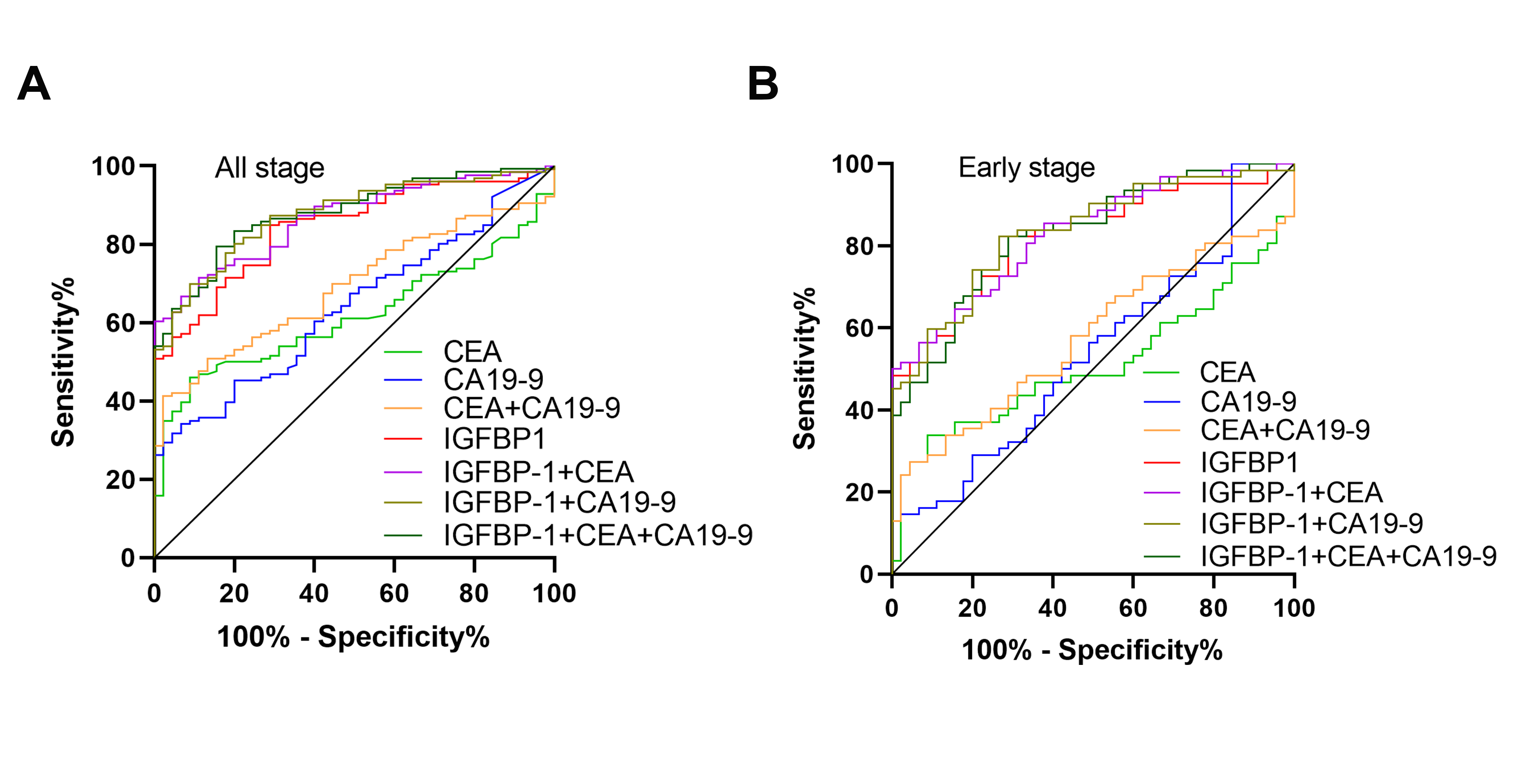

Supplement: Supplementary file 1 — Supplementary Information. [file 41598_2024_52220_MOESM1_ESM.zip › Supplementary data/supplementary figures/Supplementary Figure S2.tif]

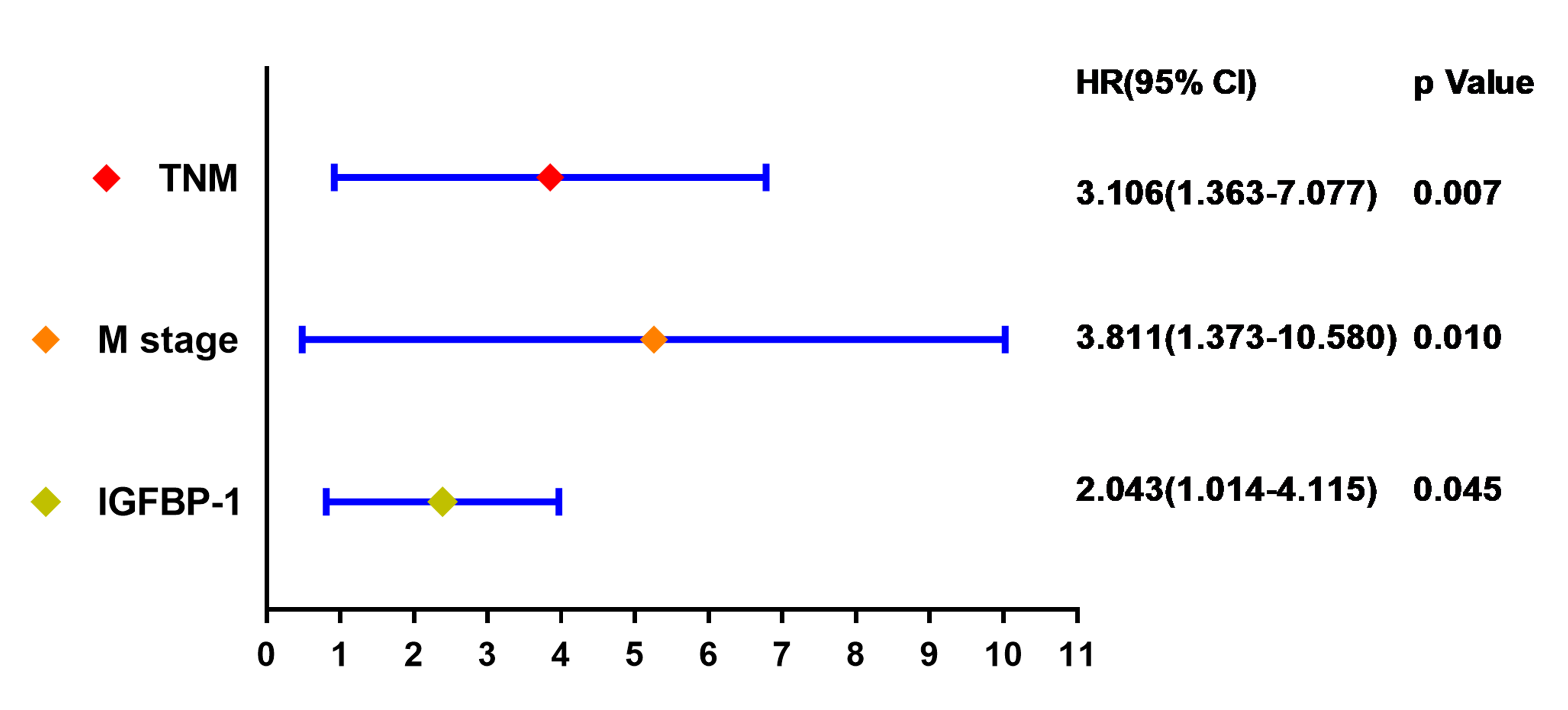

Supplement: Supplementary file 1 — Supplementary Information. [file 41598_2024_52220_MOESM1_ESM.zip › Supplementary data/supplementary figures/Supplementary Figure S3.tif]

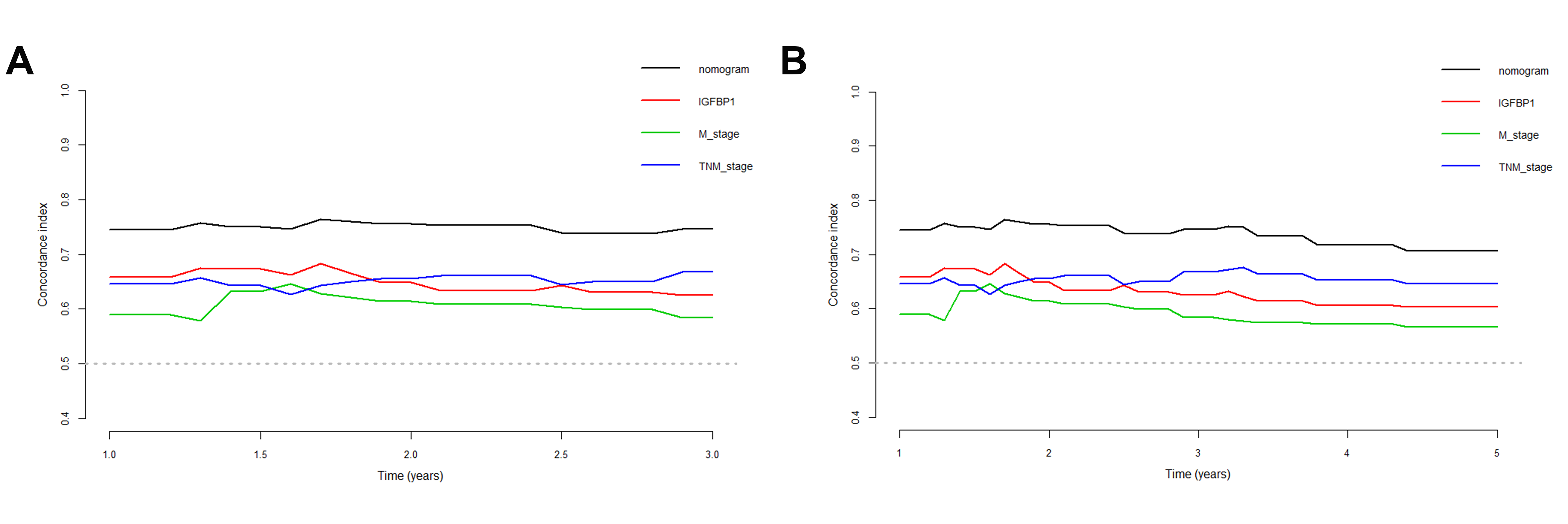

Supplement: Supplementary file 1 — Supplementary Information. [file 41598_2024_52220_MOESM1_ESM.zip › Supplementary data/supplementary figures/Supplementary Figure S4.tif]
